# Water supply, sanitation and hygiene interventions and childhood diarrhea in Kersa and Omo Nada districts of Jimma Zone, Ethiopia: a comparative cross-sectional study

**DOI:** 10.1186/s41043-019-0205-1

**Published:** 2019-12-13

**Authors:** Negasa Eshete Soboksa, Abebe Beyene Hailu, Sirak Robele Gari, Bezatu Mengistie Alemu

**Affiliations:** 1 Ethiopian Institute of Water Resources, Addis Ababa University, Addis Ababa, Ethiopia; 2 Department of Environmental Health Sciences, Jimma University, Jimma, Ethiopia; 3 College of Health and Medical Sciences, Haramaya University, Harar, Ethiopia

**Keywords:** Water supply, Sanitation, Hygiene, Intervention, Childhood diarrhea, CLTS kebeles

## Abstract

**Background:**

Diarrhea is a major public health problem that disproportionately affects children in developing countries, including Ethiopia. Most of the diseases can be prevented through safe drinking water supply and provision of basic sanitation and hygiene. However, there is a paucity of information on childhood diarrhea related to interventions in kebeles (smallest administrative structure) where community-led total sanitation (CLTS) implemented and not implemented (non-CLTS). Thus, the aim of this study was to assess and compare the association of water supply, sanitation and hygiene interventions, and childhood diarrhea in CLTS implemented and non-implemented kebeles.

**Method:**

A comparative cross-sectional study was conducted in Kersa and Omo Nada districts of Jimma Zone, Ethiopia from July 22 to August 9, 2018. Systematically selected 756 households with under-5 children were included in the study. Data were collected through interview using structured questionnaires. Water samples were collected in nonreactive borosilicate glass bottles. The binary logistic regression model was used; variables with a *p* value < 0.05 were considered as significantly associated with childhood diarrhea.

**Results:**

The prevalence of childhood diarrhea in the past 2 weeks was 17.7% (95% CI: 13.9–21.5) in CLTS kebeles and 22.0% (95% CI: 17.8–26.2) in non-CLTS kebeles. The occurrence of childhood diarrhea, increased among children whose families did not treat drinking water at home compared to those who treated in both CLTS (AOR = 2.35; 95% CI: 1.02–05.98) and non-CLTS (AOR = 1.98; 95% CI: 0.82–4.78) kebeles. About 96% of households in CLTS and 91% of households in non-CLTS kebeles had pit latrine with and without superstructure. Children from families that used water and soap to wash their hands were 76% less likely to have diarrhea in CLTS kebeles (AOR = 0.76; 95% CI: 0.31–1.88) and 54% less likely to have diarrhea in non-CLTS kebeles (AOR = 0.54; 95% CI: 0.17–1.72) when compared to children from families who used only water. The odds of having diarrhea was 1.63 times higher among children whose families live in CLTS non-implemented kebeles compared to those children whose families live in CLTS implemented kebeles (AOR = 1.63; 95% CI: 0.98–2.68).

**Conclusions:**

No significant difference was observed in the prevalence of childhood diarrhea between CLTS and non-CLTS kebeles.

## Background

Diarrhea is a major public health burden and is disproportionately affecting children in developing countries. It is the second leading cause of death in under-5 years old and responsible for killing around 525,000 children every year [1]. The disease is one of the five leading causes contributing to disability-adjusted life years (DALYs) among communicable, maternal, neonatal, and nutritional diseases (CMNNDs) in 2017 and had 76.9 million risk attributable to DALYs [2]. Children living in poor or remote communities are the most at risk and dying from these preventable diseases because effective interventions are not provided equitably across all communities [3].

Globally, around 2.4 million deaths (4.2% of all deaths) could be prevented annually if everyone practiced appropriate hygiene and had good, reliable sanitation and drinking water [4]. An estimated 88% of all child deaths as a result of disease may be prevented through improvements in water supply, sanitation and hygiene [5]. Previous systematic reviews and meta-analysis findings indicated that adequate water, basic sanitation, and hygiene interventions were associated with the reduction of diarrheal disease. For instance, point-of-use water treatment with chlorine reduces the risk of diarrhea by 25%–58% [6–9], improved sanitation can reduce diarrheal diseases by 32%–37% [10–12], and hand washing promotion reduces incidence of diarrhea by 30% [13]. A study done in Malawi also indicated that children living in families who use good-quality water supplies and latrines experience 20% less diarrhea risk [14].

The health benefits of improved sanitation were more pronounced than improved water supply [15]. To improve sanitation-related problems, various approaches have been implemented by government and nongovernmental organizations. For instance, the community-led total sanitation (CLTS), pioneered by Dr. Kamal Kar, is one of the approaches implemented to reduce open defecation and improve hygiene and sanitation practices. The approach started in different parts of developing countries by governments and NGOs to end open defecation. Study findings showed that CLTS was an important approach for increasing latrine ownerships and utilization rate [16–20]. It was also important for the reduction of childhood diarrhea [16]. But studies done in Mali and India showed that no differences were observed in terms of diarrhea prevalence among children in CLTS and non-CLTS villages [20, 21]. A recent mixed-method systematic review report also provided evidence for the need to consider CLTS as part of a larger water supply, sanitation, and hygiene (WASH) strategy rather than as a singular solution to changing sanitation behavior [18].

In Ethiopia the program began in different parts of the country to improve hygiene and sanitation practices. However, there is a paucity of information on water supply, sanitation, and hygiene interventions and childhood diarrhea among communities living in program implemented and non-implemented kebeles. Therefore, the aim of this study was to assess and compare the association of water supply, sanitation and hygiene interventions with childhood diarrhea in the CLTS implemented and non- implemented kebeles in the two selected districts in Jimma Zone, Ethiopia. The results of this study could help the government, nongovernmental organizations, and communities to design water supply, sanitation, and hygiene interventions like CLTS approaches to prevent/mitigate childhood diarrhea. This study should also assist the government in the journey to achieve the Sustainable Development Goal 6 which is “ensuring universal access to safe and affordable drinking water for all by 2030 and end open defecation by 2030.”

## Methods and Materials

### Study Setting

**Fig. 1:**
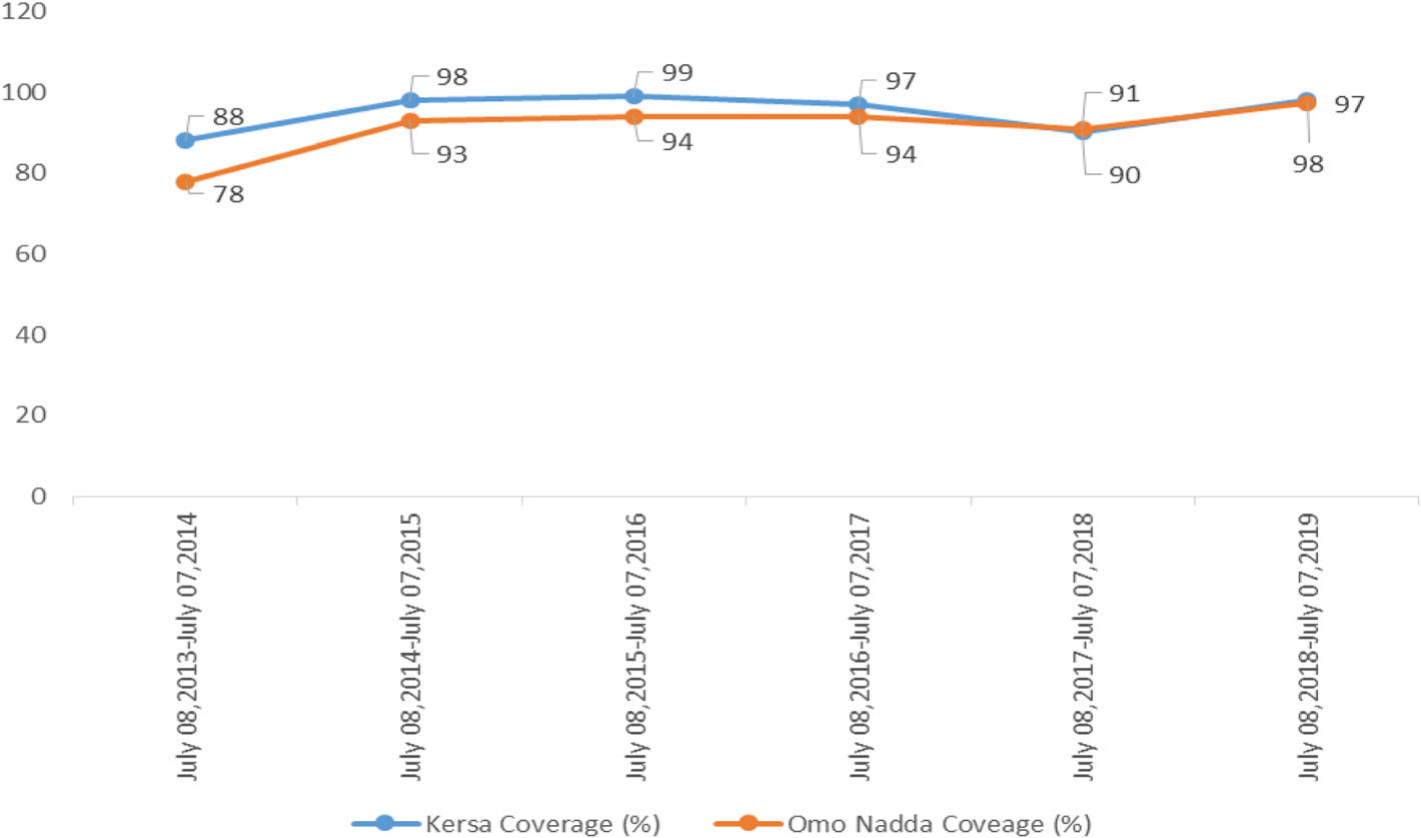
Yearly trends of all types of latrine coverage of Kersa and Omo Nada districts of Jimma Zone, Ethiopia from July 8, 2013, to July 7, 2019

The study was conducted in two selected districts (Kersa and Omo Nada) of Jimma Zone, Oromia Regional State, Ethiopia. The Zonal capital, Jimma Town, is located 357 km away from Addis Ababa in southwest Ethiopia. The zone extends between 7013′–8056′ North latitudes and 35049′–38038′ East longitudes. The altitude of these districts ranges from 1740 to 2660 m above sea level. Agriculture is the major source of economy, and it includes mainly the growing of coffee and cattle rearing. According to Jimma Zone Health Office 2011 Ethiopian Fiscal Year the population of Kersa and Omo Nada were 227,959 and 208,517, respectively. Of this population, about 81.65% residents of the Kersa district and 71.7% residents of the Omo Nada district rely on improved drinking water sources in 2018. In this year, the improved latrine coverage of the districts was 40% for Kersa and 39% for Omo Nada [22]. CLTS and hygiene approach implementation started in the Kersa district by Plan Ethiopia in July 2008. The approach concentrated on empowering local people to analyze the extent and risk of environmental pollution caused by open defecation and to construct toilets without any external subsidies. After implementation, all the households in villages constructed simple pit latrines of their own, some with slabs and covers, superstructures, and hand-washing facilities [23]. Now all rural kebeles of Kersa and limited kebeles of Omo Nada districts are declared as open defecation free. Figure 1 shows the yearly trends of all types of latrine in Kersa and Omo Nada districts after CLTS implementation started in the area.

### Study design, period, and population

Community-based cross-sectional study design was used from June 22 to August 9, 2018. All households having under-5-year-old children living in the CLTS implemented kebeles by the facilitating of Plan International Ethiopia, heath extension worker, and kebele leader; and verified kebeles served as sources of CLTS respondents, whereas all households having under-5-year-old children living in the kebeles were CLTS not implemented served as sources of respondents of non-CLTS kebeles. Respondents were those members of households who are responsible for general household responsibilities (mostly mothers).

### Sample size determination and sampling procedure

The sample size was determined by double population proportion formula, assuming the proportion of childhood diarrheal diseases at CLTS kebeles (*P*_*1*_) to be 15% (from study done in the Diretiyara district of Eastern Ethiopia) [19] and in non-CLTS kebeles (*P*_*2*_) to be 37% [24], 95% level of confidence (1.96), 80% power, and 10% non-response rate. This yielded a total of 189 households. Then after multiplying by 2 for design effect, 378 households were included in the study for interview at each site. Water sample was collected from 10% (38) of these households.

Before data collection, three CLTS kebeles and three non-CLTS kebeles were randomly selected by a lottery method from Kersa and Omo Nada districts, respectively. Then all the households with under-5 children were identified by house-to-house survey, and sample size for each kebeles was proportionally allocated. Finally, systematically selected households who have under-5 children were included in the study.

### Data collection methods

Data were collected from study respondents on sources of water for domestic uses, water storage practices, household water treatment techniques, and hygienic and sanitation practices. Respondents were also asked about their experiences with diarrhea in their households. The wealth status of the households was computed from the households’ asset ownership and housing characteristics using principal component analysis (PCA) [25] and categorized in poor, middle, and rich relatively.

Samples of water were collected in cleaned, rinsed, and sterilized nonreactive borosilicate glass bottles. Physicochemical analysis like pH was done in situ. For bacteriological analysis, the samples were immediately transported in ice-packed cooler boxes to the laboratory of Jimma University Environmental Health department. In the laboratory, samples were analyzed for indicator bacteria. To determine the degree of contamination, total coliforms and *E. coli* groups were determined by using membrane filtration technique as outlined by the APHA/AWWA/WEF [26]. This technique involves filtering water through a membrane and then incubating this membrane in m-lauryl sulfate broth at 36°C for total coliform and 44°C for *Escherichia coli*. After 24-h incubation, the yellow colonies formed were counted as total coliforms and *E. coli*. Then, the results were calculated and expressed in colony-forming unit (CFU) per 100-ml sample.

### Data quality management

To maintain data quality, data collectors and supervisors were trained intensively. The questionnaire was adapted from the WHO/UNICEF Joint Monitoring Program for Water Supply, Sanitation, and Hygiene 2017 core questions on water, sanitation, and hygiene for household surveys [27] and other literatures. The questionnaire was used after pre-test. Two supervisors followed and checked data collection processes. The necessary modifications were made on the spot, when necessary. The water sample collecting tools were sterilized, and the samples were immediately placed into a tight insulated box containing molten ice. Time between sample collection and analysis was 6 h. The investigators were also following the overall data collection procedures.

### Statistical analysis

The collected data were checked for completeness and consistency by the principal investigator and entered using EpiData version 3.1, which was exported to SPSS version 24.0 for analysis. To observe the association between dependent and independent variables, the presence of diarrhea diseases in the past 2 weeks was considered as outcome variables. Socioeconomic factors, drinking water handling, and sanitation and hygiene practices of mothers/caregivers were considered as predictor variables. Descriptive statistics for the study variables were computed and presented in tables. Binary and multivariable logistic regressions models were used to identify the study variables associated with childhood diarrheal diseases. All variables with *p* value < 0.25 in binary logistic regression analysis were entered into the multivariable logistic regression model. Those variables with *p* value < 0.05 in multivariable logistic regression model were considered as associated factors for childhood diarrheal diseases. Both crude and adjusted odd ratios with a 95% confidence interval were calculated to assess level of significance. Tables and figures were used to organize and present the data.

## Results

### Socio-demographic characteristics

Out of 756 study participants, 378 were from CLTS kebeles, and the remaining were from non-CLTS kebeles. The mean age of the respondents was 30.02 ± 6 years for CLTS kebeles and 31.60 ± 8 years for non-CLTS kebeles. About 67.7% of the respondents of CLTS kebeles and 70.4% respondent of non-CLTS kebels were females. With regard to the religions of the respondents, 87.6% of the respondents in CLTS and 99.2% in non-CLTS kebeles were Muslim. About 69.3% of respondents of CLTS and 74.1% of non-CLTS kebeles were living in households with more than five members. Concerning the educational status of the respondents, 48.4% of CLTS and 49.5% of non-CLTS kebeles have completed primary school. The prevalence of childhood diarrhea in the preceding 2 weeks was 17.7% (95% CI: 13.9–21.5) in CLTS kebeles and 22.0% (95% CI: 17.8–26.2) in non-CLTS kebeles. Of these, 58.8% in CLTS kebeles and 44.6% in non-CLTS kebeles were male (Table 1).

**Table 1:** Socio-demographic characteristics of the study participants in Kersa and Omo Nadda districts of Jimma Zone, Ethiopia

Variables		CLTS kebeles		Non-CLTS kebeles	
		Frequency	Percent	Frequency	Percent
Sex of respondent	Female	256	67.7	266	70.4
	Male	122	32.3	112	29.6
Religion	Muslim	331	87.6	375	99.2
	Orthodox	40	10.6	3	0.8
	Protestant	7	1.9	−	−
Educational status of the respondent	Illiterate	143	37.8	145	38.4
	Primary	183	48.4	187	49.5
	Secondary	44	11.6	42	11.1
	College/university	8	2.1	4	1.1
Family size	< 5	116	30.7	105	27.8
	≥ 5	262	69.3	273	72.2
Number of under-5s	1	296	78.3	223	59.0
	≥ 2	82	21.7	155	41.0
Presence of diarrhea in the past 2 weeks	Present	67	17.7	83	22
	Absent	311	82.3	285	78
Sex of children	Male	222	58.8	169	44.6
	Female	156	41.2	209	55.4
Wealth index quintile	Poor	127	33.6	189	50.0
	Medium	137	36.2	131	34.7
	Rich	114	30.2	58	15.3

### Drinking water-related characteristics

About 88.9% of respondents in CLTS households and 78.0% of non-CLTS households collect water mainly from protected sources like spring, wells, and public fountains. About 97.1% households of CLTS kebeles and 98.9% households of non-CLTS kebeles collect drinking water by Jerri can. The drinking water containers of the majority of the households were placed on the floor and not covered properly. About 4.5% households of CLTS kebeles and 9.3% households of non-CLTS kebeles informed us that they drew drinking water from storage by dipping glasses. About 28.8% of households of CLTS and 77.3% of households of non-CLTS village believed that the water tariff was affordable (Table 2).

**Table 2:** Drinking water sources and handling practices of the study participants in Kersa and Omo Nadda districts of Jimma Zone, Ethiopia

Variables		CLTS kebeles		Non-CLTS kebeles	
		Frequency	Percent	Frequency	Percent
Main source of drinking water	Protected sources	336	88.9	295	78.0
	Unprotected sources	42	11.1	83	22.0
Alternative water sources	Harvesting rain water	31	8.2	54	14.3
	Unprotected well	232	61.4	96	25.4
	River/unprotected	31	8.2	210	55.6
	Other (specify)	84	22.2	18	4.8
Average daily water consumption (l)	< 12	139	36.8	218	57.7
	12–24	206	54.5	159	42.1
	≥ 25	33	8.7	1	0.3
Approximate distance of water sources from your home (km)	≤ 1	357	94.4	318	84.1
	> 1	21	5.6	60	15.9
Time taken to fetch water (min,)	<30	363	96.0	318	84.1
	≥ 30	15	4.0	60	15.9
Container used to collect water from sources	Jerri cans	367	97.1	374	98.9
	Clay pots	9	2.4	1	0.3
	Pails	2	0.5	3	0.8
Drinking water containers covered properly	No	39	10.3	20	5.3
	Yes	339	89.7	358	94.7
Drinking water storage containers placed	On the floor	352	93.1	360	95.2
	Elevated above the floor	26	6.9	18	4.8
Cleaning water containers regularly before filling drinking water	Yes	336	88.9	369	97.6
	No	42	11.1	9	2.4
Water taken from the drinking water containers	Pouring	357	94.4	329	87.0
	Dipping glass with fingers	17	4.5	35	9.3
	Container has spigot or tap	4	1.1	14	3.7
Cost affordable	Yes	109	28.8	245	77.3
	No	269	71.2	72	22.7

**Fig. 2:**
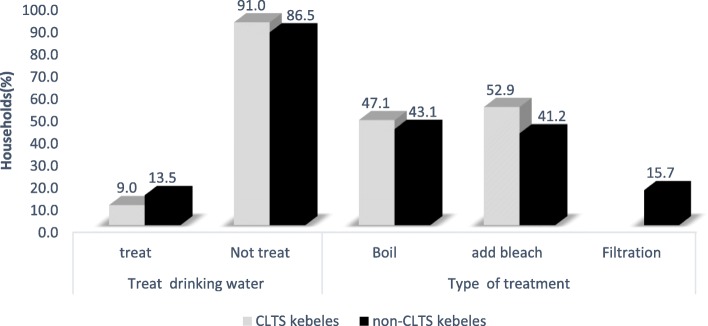
Drinking water treatment practices of households in Kersa and Omo Nadda districts of Jimma Zone, Ethiopia

Of 378 households interviewed, only about 9% households living in CLTS kebeles and 13.5% households living in non-CLTS kebeles treat their drinking water. Of these households, about 47.1% living in CLTS and 43.1% living in non-CLTS kebeles treat drinking water by boiling (Fig. 2).

### Fecal contamination of stored water in household

The pH of sampled water was 6.48 ± 0.35 and 7.03 ± 0.76 in CLTS and non-CLTS kebeles. The mean *E. coli* contamination of water at household level was 120 CFU/100 ml in CLTS and 270 CFU/100 ml in non-CLTS kebeles, respectively. From the total collected water samples, about 34.2% of sampled water from CLTS and 26.3% sampled water from non-CLTS households were free from *Escherichia coli*, whereas all water samples collected from both CLTS and non-CLTS kebeles and analyzed for total coliforms showed bacterial colonies.

### Sanitation and hygiene characteristics

About 96% households in CLTS kebeles and 91% non-CLTS kebeles had pit latrine with and without superstructures. Around 69% of households living in CLTS and 74.3% households in non-CLTS kebeles reported continuous usage of latrines. Of those who own latrines, only 4.9% households of CLTS and 1.5% households of non-CLTS kebeles shared latrines with others. Of the available latrines, about 74% in CLTS and 72% in non-CLTS kebeles do not have hand washing facilities. According to information gathered from the communities, all households of CLTS and non-CLTS kebeles prepared new latrines by covering the old ones with soil when their latrines were full of fecal sludge. There were feces in the compounds of 2.1% of households of CLTS and 86.5% of the households of non-CLTS kebeles during the visit (Table 3).

**Table 3:** Sanitation and hygiene practices of study participants in Kersa and Omo Nadda districts of Jimma Zone, Ethiopia

Variables		CLTS kebeles		Non-CLTS kebeles	
		Frequency	Percent	Frequency	Percent
Availability of latrine	Yes	364	96.3	344	91.0
	No	14	3.7	34	9.0
Type of latrine	Pit latrine with super structure	252	66.7	238	63.0
	Pit latrine without super structure	112	29.6	106	28.0
Latrine utilization	Mostly	103	27.2	63	16.7
	Always	261	69.0	281	74.3
Share latrine with other households	Yes	18	4.9	5	1.5
	No	346	95.1	339	98.5
Hand washing facilities near the toilet	yes	95	26.1	96	27.9
	No	269	73.9	248	72.1
Fecal sludge management	Preparing new by covering the old with soil	364	100	334	100
Clean latrine facility regularly	Yes	285	75.4	266	77.3
	No	79	20.9	78	22.7
Place of defecation in the absence of latrine	Open field			28	82.4
	Communal latrine	14	92.9	4	11.8
	others	1	7.1	2	5.9
Separate toilet facility for children	yes	35	9.3	67	19.5
	no	329	87.0	277	80.5
Anal cleanse material after defecation	Washes with water	325	86.0	274	72.5
	Wipe with paper	35	9.3	48	12.7
	Leaf	18	4.8	56	14.8
Observation of feces in the compound	Yes	8	2.1	327	86.5
	No	370	97.9	51	13.5
Hand washing before eating	Yes	374	98.9	377	99.7
	No	4	1.1	1	0.3
Hand washing before preparing food	Yes	274	72.5	375	99.2
	No	104	27.5	3	0.8
Hand washing before feeding a child	Yes	271	71.7	371	98.1
	No	107	28.3	7	1.9
Hand washing after defecation	Yes	336	88.9	352	93.1
	No	42	11.1	26	6.9
Hand washing after cleaning a baby’s bottom	Yes	272	72.0	359	95.0
	No	106	28.0	19	5.0
Cleansing materials used to wash hands	Water and soap	290	76.7	279	73.8
	Water and ash	21	5.6	39	10.3
	Water only	67	17.7	60	15.9

Of 378 households interviewed in CLTS kebeles, about 17.7% reported that they wash their hands with water only, whereas 76.7% washed their hands with water and soap. In non-CLTS kebeles, about 15.9% of the respondents used only water and 78.8% used water and soap to wash their hands (Table 3).

**Fig. 3:**
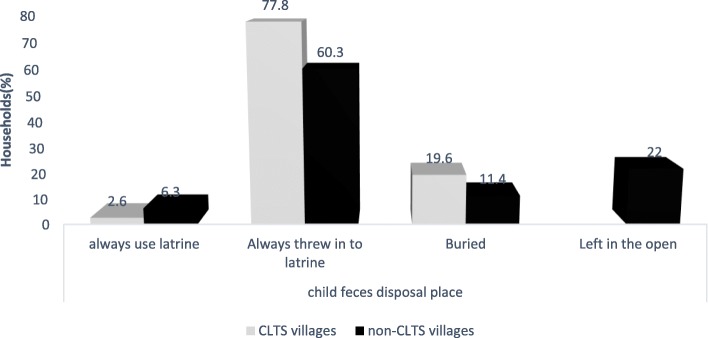
Child feces disposal practices of study participants in Kersa and Omo Nadda districts of Jimma Zone, Ethiopia

About 77.8% of respondents of CLTS and about 60.3% respondents of non-CLTS kebeles always threw child feces into the latrine. There were no respondents that throw child feces on the open field in CLTS kebeles, but about 22% of the non-CLTS kebeles respondents did that (Fig. 3).

### Factors associated with childhood diarrhea

After computing bivariate analysis, selected variables were further examined by multivariable logistic model to see their relative effects on the presence of childhood diarrheal diseases. Family size, wealth status, drinking water treatment at home, and hand washing after defecation were significantly associated with childhood diarrhea in CLTS kebeles, but in the non-CLTS kebeles family size, number of under-5 children in the households, water taking from the storage container, regular cleaning of latrines, and anal cleansing material used were significantly associated with childhood diarrhea (Table 4).

**Table 4:** Multivariable analysis of factors associated with childhood diarrhea in Kersa and Omo Nadda districts of Jimma Zone, Ethiopia

Variables		CLTS kebeles				Non-CLTS kebeles			
		Diarrhea		Crude OR (95 % CI)	Adjusted OR (95 % CI)	Diarrhea		Crude OR (95 % CI)	Adjusted OR (95 % CI)
		Yes	No			Yes	No		
		No (%)	No (%)			No (%)	No (%)		
Sex of respondent	Female	41(16)	215(84)	1.42(0.82–2.45)	1.88(0.95–3.74)	70(26.3)	196(73.7)	0.37(0.19–0.69)*	0.69(0.29–1.65)
	Male	26(21.3)	96(78.7)	1	1	13(11.6)	99(88.4)	1	1
Family Size	< 5	17(14.7)	99(85.3)	1	1	25(23.8)	80(76.2)	1	1
	≥ 5	50(19.1)	212(80.9)	0.72(0.40–1.33)	0.74(0.38–1.46)	58(21.2)	215(78.8)	1.16(0.68–1.98)	2.93(1.32–6.49)*
Number of under-fives	1	60(20.3)	236(79.7)	1	1	54(24.2)	169(75.8)		
	≥ 2	7(8.5)	75(91.5)	2.72(1.19–6.21)*	3.36(1.35–8.34)*	29(18.7)	126(81.3)	1.39(0.84–2.30)	0.42(0.19–0.92)*
Education status of the respondent	Illiterate	24(16.8)	119(83.2)	1	1	18(12.4)	127(87.6)	1	1
	Primary	31(16.9)	152(83.4)	0.99(0.55–1.77)	1.09(0.55–2.15)	39(20.9)	148(79.1)	0.54(0.29–0.98)*	0.56(0.25–1.28)
	Secondary	9(20.5)	35(79.5)	0.78(0.33–1.84)	0.97(0.37–2.57)	26(61.9)	16(38.1)	0.09(0.04–0.19)*	1.26(0.25–6.26)
	College/university	3(37.5)	5(62.5)	0.34(0.08–1.50)	0.24(0.04–1.34)	0.00%	4(100)		
Wealth status	Poor	24(18.9)	103(81.1)	0.55(0.27–1.15)	0.48(0.21–1.12)	29(15.3)	160(84.7)	1.29(0.60–2.78)	1.57(0.51–4.82)
	Medium	30(21.9)	107(78.1)	0.46(0.23–0.93)*	0.33(0.15–0.73)*	43(32.8)	88(67.2)	0.48(0.23–1.02)	1.04(0.32–3.42)
	Rich	13(11.4)	101(88.6)	1	1	11(19.0)	47(81.0)	1	1
Average daily water consumption (liters)	<12	31(22.3)	108(77.7)	1	1	34(15.6)	184(84.4)	1	1
	12-24	32(15.5)	174(84.5)	1.56(0.90–2.70)	1.47(0.75–2.88)	48(30.2)	111(69.8)	0.43(0.26–0.70)*	0.87(0.41–1.87)
	≥ 25	4(12.1)	29(87.9)	2.08(0.68–6.37)	1.87(0.52–6.67)	1(100)	−	−	−
Water taken from the drinking water containers	Pouring	65(18.2)	292(81.8)	1	1	72(21.9)	257(78.1)	1	1
	Dipping glass with fingers	2(11.8)	15(88.2)	1.65(0.37–7.48)	1.48(0.29–7.41)	8(22.9)	27(77.1)	1.03(0.23–3.78)	0.31(0.09–0.97)*
	Container has spigot or tap	-	4(100)	_	_	3(21.3)	11(78.6)	0.95(0.41–2.17)	0.79(0.12–5.10)
Treat drinking water	Yes	10(29.4)	24(70.6)	1	1	19(23.8)	61 (76.2)	1	1
	No	57(16.6)	287(83.4)	2.10(1.01-4.62)*	2.35(1.02–5.98)*	64(21.5)	234(78.5)	1.39(0.84–2.30)	1.98(0.82–4.78)
Type of latrine	pit latrine with super structure	62(18.3)	214(83.6)	0.92(0.51–1.65)	0.98(0.50–1.92)	38(16.0)	200(84.0)	0.93(0.49–1.77)	0.90(0.32–1.71)
	pit latrine without super structure	19(17.0)	93(83.0)	1	1	16(15.10	90(84.9)	1	1
Clean latrine regularly	yes	53(18.6)	232(81.4)	0.78(0.39–1.55)	0.91(0.41–2.02)	51(19.2)	215(80.8)	0.17(0.05–0.56)*	0.14(0.04–0.54)*
	no	12(15.2)	64(84.8)	1	1	3(3.8)	75(96.2)	1	1
Child feces disposal place	Use latrine	2(20.0)	8(80.0)	1	1	10(41.7)	14(58.3)	1	1
	thrown in to latrine	51(17.3)	243(82.7)	1.19(0.25–5.78)	1.12(0.16–7.75)	29(12.7)	199(87.3)	4.09(1.99–12.06)*	2.74(0.25–9.59)
	Buried	14(18.9)	60(81.1)	1.07(0.21–5.61)	0.64(0.08–4.89)	13(30.2)	30(69.8)	1.65(0.58–4.67)	0.66(0.05–8.74)
	Left in the open	-	-	-		31(37.3)	52(62.7)	1.21(0.48–3.02)	0.49(0.04–6.53)
Anal cleanse material after defecation	Washes with water	55(16.9)	270(83.1)	1	1	66(24.1)	208(75.9)	1	1
	Wipe with paper	5(14.3)	30(85.7)	1.19(0.44–3.23)	1.30(0.44–3.87)	7(14.6)	41(85.4)	1.86(0.80–4.34)	2.78(1.41–8.34)*
	Leaf	7(38.9)	11(61.1)	1.15(0.42–3.12)	1.02(0.33–3.15)	10(17.9)	46(82.1)	1.46(0.70–3.05)	4.58(2.01–16.47)*
Hand washing facilities near the latrine	Available	21(22.1)	74(77.9)	1	1	18(18.8)	78(81.3)	1	1
	Not available	44(16.4)	225(83.6)	145(0.81–2.60)	1.50(0.74–3.04)	36(14.5)	212(85.5)	1.31(0.71–2.45)	1.33(0.59–3.01)
Hand washing before food preparing	Yes	57(20.8)	217(79.2)	0.41(0.20–0.83)*	0.94(0.33–2.68)	82(22.1)	289(77.9)	0.59(0.07–4.95)	−
	No	10(9.6)	94(90.4)	1	1	1(14.3)	6(85.7)	1	1
Hand washing after defecation	Yes	65(19.3)	271(80.7)	0.21(0.05–0.88)*	0.14(0.03–0.67)*	79(22.4)	273(77.6)	0.63(0.21–1.88)	1.44(0.28–7.24)
	No	2(4.8)	40(95.2)	1	1	4(15.4)	22(84.6)	1	1
Cleansing materials used to wash hands	Water and soap	55(19.0)	235(81.0)	0.66(0.31–1.42)	0.76(0.31–1.88)	53(19.0)	226(81.0)	0.75(0.35–1.62)	0.54(0.17–1.72)
	Water and ash	3(14.3)	18(85.7)	0.92(0.23–3.81)	0.88(0.17–4.45)	21(53.8)	18(46.2)	0.15(0.06–0.39)*	−
	water only	9(13.4)	58(86.6)	1	1	9(15.0)	51(85.0)	1	1

*Significant at *p* < 0.05

The occurrence of childhood diarrhea was 2.93 times higher among children living in households with 5 or more members than those with a family size of less than five members in non-CLTS kebeles (AOR = 2.93; 95% CI: 1.32–6.49). But, in CLTS kebeles, the occurrence of childhood diarrhea was reduced by 26% among children living in households with family size greater than or equal to five compared to children living in households with a size of less than five (AOR = 0.74; 95% CI: 0.38–1.46). The absence of point-of-use drinking water treatment increased the occurrence of diarrheal diseases in both CLTS (AOR = 2.35; 95% CI: 1.02–05.98) and non-CLTS (AOR = 1.98; 95% CI: 0.82–4.78) kebeles. On the contrary, the likelihood of childhood diarrhea occurrence was less among households who clean their latrine regularly in both CLTS (OR = 0.91; 95% CI: 0.41–2.02) and non-CLTS (AOR = 0.14; 95% CI: 0.04–0.54) kebeles. Children from families who used water and soap to wash their hands were less likely to having diarrhea in CLTS kebeles (AOR = 0.76; 95% CI: 0.31–1.88) and non-CLTS kebeles (AOR = 0.54; 95% CI: 0.17–1.72) when compared to children from families who used only water (Table 4).

Table 5 shows the multivariable regression analysis of combined factors with childhood diarrhea by assuming CLTS as predictor variable to see whether CLTS implementation significantly reduces childhood diarrhea. In this table, wealth status, drinking water treatment, regular cleaning of latrine, anal cleansing material after defecation, and hand washing before preparing food and after defecation have not statistically significant association with childhood diarrhea, whereas the remaining predictors like sex of the respondent, family size, number of under-5, education status, average daily water consumption (liters), water taken from the drinking water containers, type of latrine, child feces disposal place, hand washing with water and soap, and CLTS status were not statistically significantly associated with childhood diarrhea. The odds of having diarrhea was 1.63 times higher among children whose family live in non-CLTS kebeles compared to those children whose family live in CLTS kebeles (AOR = 1.63; 95% CI: 0.98–2.68) (Table 5).

**Table 5:** Multivariable regression analysis of CLTS status and other factors with childhood diarrhea in Kersa and Omo Nadda districts of Jimma Zone, Ethiopia

Variables		Diarrhea		Adjusted OR (95 % CI)
		Yes	No	
		No (%)	No (%)	
Sex of respondent	Female	111(21.3)	411(78.7)	0.91(0.56–1.46)
	Male	39(16.7)	195(83.3)	1
Family Size	<5	42(19.0)	179(81.0)	1
	≥ 5	108(20.2)	427(79.8)	1.27(0.79–2.05)
Number of under-5s	1	114(22.0)	405(78.0)	1
	≥ 2	36(15.2)	201(84.8)	1.25(0.76–2.06)
Education status of the respondent	Illiterate	42(14.6)	246(85.4)	1
	Primary	70(18.9)	300(81.1)	0.79(0.49–1.26)
	Secondary	35(40.7)	51(59.3)	0.78(0.36–1.69)
	College/university	3(25.0)	9(75.0)	0.39(0.09–1.66)
Wealth status	Poor	53(16.8)	263(83.2)	0.80(0.43–1.49)
	Medium	73(27.2)	195(72.8)	0.46(0.25–0.84)*
	Rich	24(14.0)	148(86.0)	1
Average daily water consumption (liters)	<12	65(18.2)	292(81.8)	1
	12-24	80(21.9)	285(78.1)	1.30(0.83–2.05)
	≥ 25	5(14.7)	29(85.3)	1.16(0.38–3.49)
Water taken from the drinking water containers	Pouring	137(20.0)	549(80.0)	1
	Dipping glass with fingers	10(19.2)	42(80.8)	0.90(0.41–2.00)
	Container has spigot or tap	3(16.7)	15(83.3)	1.54(0.30–7.87)
Treat drinking water	Yes	29(25.4)	85(74.6)	1
	No	121(18.8)	521(81.2)	2.13(1.21–3.74)*
Type of latrine	Pit latrine with super structure	100(19.5)	414(80.5)	0.97(0.59–1.59)
	Pit latrine without super structure	35(16.1)	183(83.9)	1
Clean latrine regularly	Yes	104(18.9)	104(81.1)	0.36(0.19–0.66)*
	No	15(9.7)	139(90.3)	1
Child feces disposal place	Use latrine	12(35.3)	22(64.7)	1
	Thrown in to latrine	80(15.3)	442(84.7)	1.05(0.26–4.23)
	Buried	27(23.1)	90(76.9)	0.38(0.09–1.65)
	Left in the open	31(37.3)	52(62.7)	0.33(0.06–1.74)
Anal cleanse material after defecation	Washes with water	121(20.2)	478(79.8)	1
	Wipe with paper	12(14.5)	71(85.5)	2.08(0.96–4.51)
	Leaf	17(23.0)	57(77.0)	3.02(1.19–7.64)*
Hand washing facilities near the latrine	Available	39(20.4)	152(79.6)	1
	Not available	80(15.5)	437(84.5)	1.28(0.79–2.09)
Hand washing before food preparing	Yes	139(21.6)	506(78.4)	0.31(0.14–0.70)*
	No	11(9.9)	100(90.1)	1
Hand washing after defecation	Yes	144(20.9)	544(79.1)	0.27(0.09–0.78)*
	No	6(8.8)	62(91.2)	1
Cleansing materials used to wash hands	Water and soap	108(19.0)	461(81.0)	0.64(0.34–1.19)
	Water and ash	24(40.0)	36(60.0)	2.23(0.57–8.75)
	water only	18(14.2)	109(85.8)	1
CLTS status	CLTS kebeles	67(17.7)	311(82.3)	1
	Non-CLTS kebeles	83(22.0)	295(78.0)	1.63(0.98–2.68)

*Significant at *p* < 0.05

## Discussion

This study showed the relations between water supply, sanitation and hygiene interventions, and the prevalence of childhood diarrhea in CLTS implemented and non-implemented kebeles in Kersa and Omo Nadda districts of Jimma Zone, Ethiopia. It was found that almost all households in CLTS and non-CLTS kebeles collected water from protected sources. But the use of protected source does not always mean safe. It might be contaminated with pathogens during transport and storage. In this study, about 34.2% of sampled water from CLTS and 26.3% sampled water from non-CLTS households were free from *Escherichia coli* and met the WHO recommended guidelines for drinking water [28]. The analysis of this study indicated that water contamination was higher in non-CLTS households than CLTS households. This finding was similar to a study done in India [29] but inconsistent with cluster-randomized controlled trial study in Mali [21]. Even though the contamination level is above the recommended value [28], CLTS implementation might be an important approach for minimization of open defecation which plays an important role for fecal contamination of water [29, 30].

This study showed that point-of-use drinking water treatment practices by households were low in both CLTS and non-CLTS kebeles. This indicates that the promotion of water treatment to improve water quality was low in both kebeles. Affordability has a significant influence on the use of water and a selection of water sources. The high cost of water can force households to use alternative sources of water of poorer quality that pose a greater risk to their health [31]. In the present study, about 71.2% households in CLTS and 22.7% households in non-CLTS kebeles reported that the fee they paid for water was not affordable.

Studies done in Ethiopia, Mali, and India showed that implementation of CLTS increased the accessibility of latrine [19–21]. The findings of this study also indicated that the latrine coverage was higher in CLTS kebeles than non-CLTS kebeles. The latrines were simple pit latrine with superstructure made of local available materials and without. The lack of hand washing facilities near latrine and the lack of soap and water are the main reasons why people do not wash their hands after defecation [32]. In the present study, about 26% of households with latrine in CLTS and 28% of households with a latrine in non-CLTS kebeles had no hand washing facilities.

Inadequate hand washing after defecation is an important source of transmission of diseases like diarrhea [33]. The practice of hand washing after defecation in the two districts was found to be better than shown by a study in Afghanistan in which 25% of households washed their hands with water and soap after defecating [34]. This difference might be due to the implementation of health extension program of Ethiopia in line with CLTS implementation.

The findings of this study showed that statistically significant difference was not observed between the CLTS kebeles and non-CLTS kebeles regarding the preceding 2-week childhood diarrhea. Similar finding was reported in Mali and India [20, 21]. But other studies done in Ethiopia revealed that the diarrhea prevalence was lower in CLTS than non-CLTS villages [16, 19, 35]. The difference might be due to variation in management of human feces of the sample community or type of study design we used.

The childhood diarrhea was statistically associated with a number of under-5 children in the households in CLTS kebeles. This is consistent with previous studies done in Ethiopia and Pakistan [36, 37]. But in non-CLTS, the odds of childhood diarrhea were lower in households with more than two children which contradicted the finding of CLTS kebeles. The difference could be attributed to socioeconomic status of the sampled community. The study indicated that wealth status was statistically associated with childhood diarrhea in CLTS kebeles and in non-CLTS kebeles. Children whose families were poor had higher odds of having childhood diarrhea even if it was not statistically significant. This study is in agreement with studies done in Ethiopia and Nigeria [36, 38]. This might be because rich families have greater opportunity to provide a good source of drinking water and use improved sanitation facilities and soap for washing purposes.

In our study, having family size greater than five was more likely to increase the odds of childhood diarrhea in the non-CLTS kebeles, whereas, in CLTS implemented kebeles, the odds of childhood diarrhea was lower. In CLTS implemented kebeles, the association between childhood diarrhea and family size had inverse relations. This might be due to good sanitation and hygiene and water handling practices of household living in the CLTS kebeles that reduced the odds of exposure to diarrhea, despite large family size.

In CLTS kebeles, it was found that the odds of having childhood diarrhea were higher among children living in families who draw water from storage containers by dipping glass with fingers compared to those taking by pouring. But in non-CLTS kebeles, on the contrary, dipping glass with finger reduces the odds of having diarrhea. It seems that either the report of the respondents in the non-CLTS kebeles might not be true, or they might use mixed method. The findings of this study indicated that the odds of having diarrhea among children living in families who treat drinking water at point-of-use was lower in CLTS and non-CLTS kebeles. This finding was in line with previously conducted studies in Nigeria [39] and in Myanmar [40] and systematic review done by Darvesh et al. [41]. But it contradicted with the study done in Afghanistan [34].

In this study, children from families who used the pit latrine with super structure were less likely to have diarrhea when compared to children from families, who used pit latrine without superstructure in both CLTS and non-CLTS kebeles. Similarly, a study done in Nigeria indicated that children in households with access to improved latrine show lower odds for diarrhea than those using unimproved sanitation [42]. This might be due to a hygienic separation of human excreta from human contact that decreased the risk of exposing to diarrheal disease infectious agents. The odds of having diarrhea has significantly decreased among children living in families who regularly clean their latrines in non-CLTS kebeles. This finding corresponds to other studies done in Vietnam and Jordan [43, 44]. But, in CLTS kebeles, the odds of childhood diarrhea were lower, but not significant in households who clean their latrine regularly. This might be due to the fact that other variables entered into the model which has a higher impact than this variable.

The study also revealed that hand washing significantly reduced the odds of childhood diarrhea among children of CLTS kebeles. But in non-CLTS kebeles, hand washing increased the odds of childhood diarrhea. This could be attributed to lesser hand washing effectiveness that created favorable conditions for reproduction instead of removal of the infectious agent, or it could be related to a lower quality of water used and the absence of soap. Hand washing with soap has been reported to reduce diarrheal morbidity by 44% [45, 46]. This study also showed that hand washing with water and soap reduced the odds of childhood diarrhea in both CLTS and non-CLTS kebeles.

This study has some limitations. Recall bias and community desirability bias may have occurred due to the individual decision of the mother regarding diarrhea and poor reporting of behavioral factors like defecation site, hand washing, and child feces disposal practices. Due to financial problem, the amount of water sampled was low, and the results were not included in the regression model.

## Conclusions

The study showed that most of the households of CLTS and non-CLTS kebeles collected water from protected sources. However, almost all water samples collected from these households were contaminated by fecal bacteria and were unsafe for human consumption. In addition, household’s water treatment practice at the point of use was still low in both CLTS and non-CLTS kebeles. In this study great difference was not observed among the two kebeles in latrine accessibility and utilization. More than one-fourth of the study households in both CLTS and non-CLTS kebeles used pit latrine without supper structure which is favorable for flies to bread and cause feco-oral diseases. Most of these latrines also do not have any kind of hand washing facilities. We found strong evidence that differentiate CLTS kebeles from non-CLTS kebeles in feces management as much more child feces were observed in non-CLTS compounds than those of CLTS. No statistically significant difference was observed in the prevalence of childhood diarrhea between CLTS kebeles and non-CLTS kebeles. Moreover, factors like family size, wealth status, drinking water treatment at home, and hand washing after defecation in CLTS kebeles and family size, number of under-5 children in a household, water taking from the storage container, regular cleaning of latrines, and anal cleansing material used in the non-CLTS kebeles were important variables for the prevention of childhood diarrhea. Therefore, effective health promotion and raising awareness of household’s on drinking water handling, regular latrine cleaning, and hand washing after defecation to prevent childhood diarrhea and to achieve Sustainable Development Goal targets in both CLTS and non-CLTS kebeles are recommended.

## Data Availability

Contact the corresponding author for data and material

## Abbreviations

AOR: Adjusted Odd Ratio
CI: Confidence interval
CLTS: Community-led total sanitation
cm: Centimeter
JMP: Joint Monitoring Program for Water and Sanitation
km: Kilometer
MDG: Millennium Development Goal
SD: Standard deviations
UNICEF: United Nations Children’s Fund
WASH: Water supply, sanitation, and hygiene
WHO: World Health Organization
